# Septate Uterus in a Girl with Rubinstein–Taybi Syndrome

**DOI:** 10.1155/2018/7878156

**Published:** 2018-04-08

**Authors:** Filipa de Castro Coelho, Sara Câmara, Inês Alves, Kathleen Brazão

**Affiliations:** ^1^Department of Gynecology and Obstetrics, Hospital Dr. Nélio Mendonça, Serviço de Saúde da Região Autónoma da Madeira, E.P.E. (SESARAM), Funchal, Portugal; ^2^Department of Radiology, Hospital Dr. Nélio Mendonça, Serviço de Saúde da Região Autónoma da Madeira, E.P.E. (SESARAM), Funchal, Portugal

## Abstract

Rubinstein–Taybi syndrome is an extremely rare plurimalformative condition that can affect any organ. However, reports regarding gynecological problems are unusual. We report the first case of a septate uterus in an adolescent with this syndrome, in agreement with the American Society for Reproductive Medicine (ASRM) and the Congenital Uterine Malformations by Expert (CUME) criteria for uterine septum. Additional studies are required to determine whether there is an increased frequency of müllerian duct anomalies with the condition. Our report extends the data on the clinical phenotype associated with Rubinstein–Taybi syndrome.

## 1. Introduction

Rubinstein–Taybi syndrome (RSTS; OMIM 180849) is an extremely rare plurimalformative condition that was first described in 1963 [1]. The syndrome is almost always a de novo occurring autosomal entity [2]. RSTS can be caused by heterozygous mutations in CREBBP gene or less commonly the EP300 gene, which are involved in several basic cellular activities including growth and differentiation. Although nearly 45% of cases have no identifiable mutation, there are no precise clinical diagnostic criteria [2, 3]. Classical features include postnatal growth retardation, microcephaly, distinctive facial characteristics, broad thumbs and big toes, and intellectual disability [4, 5]. Data on children and adults with RSTS have been extensively gathered in many areas to guide decisions in medical care of these patients. However, case reports regarding gynecologic health care in RSTS are particularly rare in the literature. Here, we report a first case of a septate uterus in a girl with RSTS. Our objectives are to highlight this unreported clinical finding and to discuss the importance of gynecological development evaluation to appropriate management and follow-up care of RSTS girls.

## 2. Case

**Figure 1:**
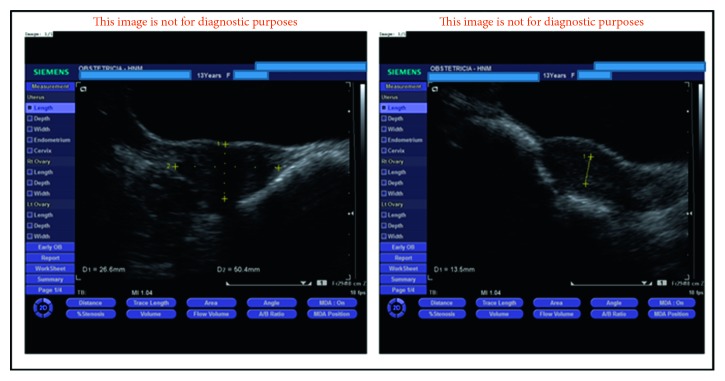
Ultrasound images showing a normal size uterus with two endometrial cavities both with a regular endometrium.

**Figure 2:**
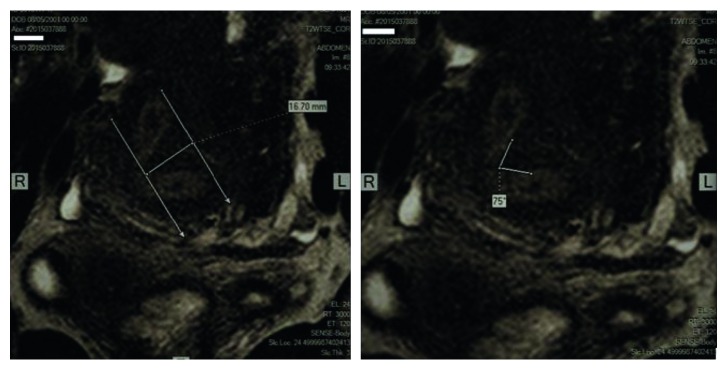
MRI and coronal view of the septate uterus. Internal indentation depth is 16.7 mm and the indentation angle is 75°, with a normal external fundal contour, and these measurements are in agreement with the definition for septate uterus by the ASRM and the CUME [7, 8].

A 13-year-old girl was referred with the complaint of spotting since menarche at age 12 years. She was known to have clinical diagnosis of RSTS with normal genetic studies. The condition affected her ability to care for herself, and she was living in a state-supported institution for children with intellectual disability since her parents' divorce. She had no history of dysmenorrhea, sexual intercourse, nor previous gynecological examination. Clinical examination at presentation was unremarkable, except an increased body mass index. Serum human chorionic gonadotropin level was within nonpregnant levels. Complete blood count, clotting screens, and thyroid function tests were in the normal ranges. Transabdominal ultrasound examination showed a normal size uterus with two endometrial cavities both with a regular endometrium with a thickness of 13.5 mm (Figure 1) and normal ovaries. MRI confirmed the presence of two endometrial cavities separated by a small midline septum without proximity to the internal os. The flat fundus with an internal indentation depth of 16.7 mm and an indentation angle of 75° was consistent with a partial septate uterus (Figure 2) (according to the Congenital Uterine Malformations by Expert (CUME) criteria and the American Society for Reproductive Medicine (ASRM) classification system of female tract congenital anomalies). Coexistent renal abnormalities were excluded.

The patient was started on cyclical progestogen for dysfunctional uterine-bleeding management. She became asymptomatic and had regular menses with normal menstrual bleeding during a 4-month follow-up period. The management of the partial septate uterus needed nothing more than reassurance. Parental consent was given and a progestogen-only implant was inserted to prevent the risk of unintended pregnancy.

## 3. Discussion

RSTS is a dominantly inherited syndrome characterized by multiple congenital anomalies and mental retardation, with an estimated birth prevalence of one in 100,000–125,000 live births. About 50% of archetypical RSTS carry deletions or mutations of CREBPP gene; mutations in EP300 gene have also been detected, but cytogenetic and molecular studies with negative results do not exclude the diagnosis [2, 3]. Most of RSTS cases are still currently diagnosed based on characteristic features [5]. Besides the classic phenotype previously described, several birth defects have been reported to occur in persons with RSTS; however, congenital uterine malformations are not typically known to be associated with the syndrome.

A septate uterus can occur during embryonic life due to defective regression of the midline septum uniting the two müllerian ducts. Uterine malformations are commonly associated with renal anomalies because of the close embryological origin of the renal and genital tracts. An interesting remark is that renal anomalies are considered a typical feature of RSTS, and all children with the syndrome should receive a baseline renal ultrasound [4, 5]. When a single septate uterus is diagnosed, urinary malformations are usually absent [6, 7], which is consistent with the current report.

Distinguishing between septate and normal/arcuate uterus, it is point of ongoing debate among experts. The risks of overdiagnosis and overtreatment of septate uterus diagnosis by the European Society of Human Reproduction and Embryology-European Society for Gynaecological Endoscopy (ESHR–ESGE) classification of müllerian duct anomalies are higher, when compared to the ASRM and CUME criteria [8, 9]. As recommended for daily practice, we classified this reported congenital uterine anomaly using the CUME reference standard (internal indentation depth ≥ 10 mm) as septate uterus, in agreement with experts' opinion from different societies in the field [8]. The recognition of the septum was also in agreement with the ASRM criteria (indentation depth > 15 mm and indentation angle < 90°) [7].

RSTS management strategies are symptomatic [2, 4]. The same reasoning should be used to congenital uterus malformations. In the present case report, expectant management was a suitable option facing the incidental finding of a müllerian anomaly: (i) our case describes a minor female with genital tract malformation; (ii) our patient was asymptomatic: as the spotting complaints could not be justified by the presence of a partial septum, she had no dysmenorrhea, and septate uterus is associated especially with obstetrical problems; and (iii) surgical repair with resection of the septum is primarily directed to improve pregnancy outcome; however, there are no randomized controlled trials confirming benefits and safety of such procedures [7].

The great majority of RSTS patients, such as the present one, have a manifest cognitive delay which itself accounts for RSTS women be considered a high-risk obstetric population. Although global mental retardation is characteristic, RSTS patients have a marked ability to establish excellent social contacts and can reproduce [2]. If a uterine malformation has been detected, the obstetric risk could be higher. Congenital anomalies of the uterus typically do not prevent conception and implantation [6]. Therefore, a critical aspect is to assess contraceptive need as initially indicated in RSTS medical guidelines [4]. To avoid unintended pregnancy, we recommended and have provided a long-acting reversible contraception, that is, an etonogestrel-containing subcutaneous implant to our adolescent patient.

Medical guidelines proposed by Wiley et al. in 2003 include gynecological evaluation of RSTS females [4], but the same is not seen in a recent proposal for update health care and follow-up care of RSTS [5]. However, the authors mention that RSTS management should be adjusted in adolescent age, and that, further investigation should also focus clinical diagnosis for refinement of guidelines [5]. Each RSTS female should have an individualized approach to gynecologic health care, given the complexity and rarity of the condition. We also suggest careful surveillance during adolescence, in order to increase the chances to uncover gynecological problems during this particular time which is already marked by great physical and mental changes. It is imperative to treat RSTS girls with particular patience and skill. Evaluation by a gynecologist knowledgeable in caring for adolescents with developmental disabilities is warranted. Early referral based on presenting symptoms allows a collaborative effort for better management and follow-up care of RSTS female patients.

RSTS is a plurimalformative syndrome that can affect any organ [5]. We describe for the first time a septate uterus in an RSTS girl. To date, there is one prior reference to a patient with RSTS who had a *bifid* uterus associated with menometrorrhagia, but details are not known [4]. Additional studies are required to determine whether there is an increased frequency of müllerian duct anomalies with the condition. Müllerian defects are not infrequently observed in malformation syndromes [5]. But many uterine anomalies are an asymptomatic incidental finding as in our report. As previously discussed, abnormal embryologic development of the uterus is frequently associated with urologic malformations [6]. It is unclear if this can be extrapolated to the females among the 52% of patients with RSTS who exhibit renal malformations [4, 5]. The underlying etiology of congenital uterine defects is not well understood [6, 10]. Research regarding RSTS etiopathogenesis is in progress [3, 5]. No cytogenetic or molecular abnormality has been detected in our patient, and therefore, no genotype/phenotype correlation can be theorized.

Ultimately, our report extends the data on the clinical phenotype associated with this extremely rare multiple congenital anomaly. With the publication of similar cases, the attention of clinicians and researchers in the field of RSTS will be brought to gynecological development evaluation, providing best practice in medical care of female patients with RSTS.
